# Into the night: camera traps reveal nocturnal activity in a presumptive diurnal primate, *Rhinopithecus brelichi*

**DOI:** 10.1007/s10329-012-0318-2

**Published:** 2012-07-29

**Authors:** Chia L. Tan, Yeqin Yang, Kefeng Niu

**Affiliations:** 1 San Diego Zoo Institute for Conservation Research, San Diego Zoo Global, 15600 San Pasqual Valley Road, Escondido, CA 92027, USA; 2 Fanjingshan National Nature Reserve Administration, Tongren, 554400 Guizhou People’s Republic of China

**Keywords:** Camera traps, *Rhinopithecus brelichi*, Diel activity pattern, Nocturnality, Fanjingshan

## Abstract

Most living primates exhibit a daytime or nighttime activity pattern. Strict diurnality is thought to be the rule among anthropoids except for owl monkeys. Here we report the diel activity pattern of an Asian colobine, the Guizhou snub-nosed monkey *Rhinopithecus brelichi*, based on a methodology that relied on using 24-h continuously operating camera traps. We conducted the study in Fanjingshan National Nature Reserve in Guizhou, China from March 22 to May 19 and from June 17 to October 14, 2011. After standardizing all time elements to a meridian-based time according to the geographic coordinates of the study site, we showed unequivocally that the monkeys, though predominantly diurnal, exhibited activity beyond daylight hours throughout the study. Specifically, their activity at night and during twilight periods suggests a complex interplay of behavioral adaptations, among others, to living in a temperate environment where day length and food resources fluctuate substantially across seasons. We contend that, under prevailing ecological conditions, so-called strictly diurnal primates may adjust their activity schedule opportunistically in order to increase energy intake. We also discuss the advantages of using camera traps in primate studies, and how the standardized use of meridian-based time by researchers would benefit comparisons of diel activity patterns among primates.

## Introduction

Camera traps have become an effective tool for studies of wildlife populations without direct observation or physically capturing animals. Use of camera traps is particularly ideal for surveying elusive animals in vast, remote areas with difficult field conditions that prohibit adequate sampling using more traditional methodologies (Kays and Slauson 2008; O’Connell et al. 2011). Beyond population surveys and monitoring, camera traps are useful in ecological and behavioral research, as images captured may reveal key information about habitat, and the behavior of species on an individual as well as group basis (Bridges and Noss 2011). Because most primate species are not cryptic, direct observation by researchers has proven to be generally effective, with behavioral data often being collected over prolonged periods of time especially following habituation of animals. Thus, camera traps appeared to offer limited applications in primate behavioral studies (e.g., Blake et al. 2010; Pebsworth et al. 2011).

Free-ranging Chinese snub-nosed monkeys (*Rhinopithecus* spp.) present huge field challenges for researchers. Elusive and difficult to track, these monkeys form supertroops of 100–400 individuals that traverse mountainous terrain, covering large home ranges that often exceed 20 km^2^ (Kirkpatrick et al. 1998; Tan et al. 2007; Grueter et al. 2008; Niu et al. 2010). We applied camera trap technology to our research on free-ranging Guizhou snub-nosed monkeys *R. brelichi*—an endangered species with a single global population of 700–800 individuals restricted to Fanjingshan in southwest China (Yang et al. 2002). The intent was to reduce the knowledge gaps impeded by brief contact time with the monkeys through direct observation. Specifically, in this study we focused on the diel (24-h) activity pattern of *R. brelichi*. Camera traps offered the advantage of uninterrupted monitoring, while minimizing disturbance caused by human observers, and eliminating the need for habituation that potentially could lead to increased poaching.

On the basis of known habits, morphological features, and phylogenetic affinity, *R. brelichi* is assumed to be exclusively diurnal (e.g., Bleisch and Xie 1998; see Kay and Kirk 2000, Table 1; Yang et al. 2002; Niu et al. 2010). Here we report our camera-trap results from an initial six-month investigation. We address the ecological implications of the observed activity pattern, and discuss the significance of our findings in conjunction with methodological improvements needed for studying this and other presumptive solely diurnal species.

## Methods

This study was conducted in Fanjingshan National Nature Reserve (FNNR) in northeast Guizhou Province, China (27°49′–28°01′N, 108°45′–48′E). FNNR contains 41,900 ha of subtropical and temperate forests with major vegetation zones graded according to elevations: evergreen broadleaf forest (<1,300 m), mixed evergreen and deciduous broadleaf forest (1,300–2,200 m), and mixed deciduous broadleaf, conifer, and scrub forest (>2,200 m) (Yang et al. 2002). The climate is seasonally distinct. Snow and freezing rain are common in winter and early spring. In 2010–2011, the mean monthly temperatures varied from −4.4 °C (January) to 21.9 °C (July), and the annual precipitation was 1,606 mm (using Onset^®^ Hobo U30/NRC Weather Station, Tan et al. unpubl. data from 1,300 m elevation). Fanjingshan is the only refuge for the focal species, *R. brelichi*. Another nonhuman primate, the Tibetan macaque *Macaca thibetana*, occurs sympatrically.

As part of a reserve-wide, biodiversity survey and an in-depth study of *R. brelichi*, we deployed 40 remote digital cameras with passive infrared sensors (i.e., camera traps) in Yangaoping, located in the northeast parcel of FNNR. Yangaoping, ranging from 800 to >2,000 m in elevation, constitutes the core habitat of *R. brelichi* (Bleisch and Xie 1998; Yang et al. 2002; see Niu et al. 2010, Fig. 1 for map). Details regarding the digital images reported here are from two camera traps, LT003 and LT004, set approximately 10 m apart in an area dominated by beech trees *Fagus lucida* (27°57′09.8–10.3″N, 108°45′22.4–24.3″E, elevation 1,650 m). The cameras (Bushnell^®^ Trophy Cam™ XLT119435c), each equipped with a 2-gigabyte Secure Digital (class 2) memory card and 8 extended-use AA batteries, were mounted below the canopy at 5–6 m above ground level. Before operating, we adjusted the infrared sensor to normal, the capture setting to the multi-image mode of 3 images per trigger, and the time lapse between triggers to 10-s intervals. We also programmed the date and time according to China Standard Time (CST). The cameras operated continuously throughout the 24-h cycle from March 22 to May 19 and from June 17 to October 14, 2011, totaling 294 trap days.

Images captured by the two camera traps were cataloged separately, then classified by species, date, and time. We evaluated whether or not an image series (in multiples of three) constituted an independent event by adopting the definition outlined by O’Brien et al. (2003) with minor modifications. Specifically, we defined an independent event as a (1) successive image series of different individuals of the same or different species, (2) successive image series of individuals of the same species captured >1 h apart, or (3) nonsuccessive image series of individuals of the same species. Because the two camera traps were deployed in the same general area, any image series of a species from the two cameras with overlapping date and time were treated as a single event.

We subsequently assigned an activity period to each independent event based on its time of occurrence. Using the definitions of the Astronomical Applications Department of the United States Naval Observatory (USNO) (http://aa.usno.navy.mil) regarding the rising and setting of the sun, we categorized the activity period of each event as:

- Twilight = occurring during periods of civil twilight (i.e., when the sun is 0–6° below the horizon) either before sunrise or after sunset;
- Daytime (or diurnal) = occurring during the period between sunrise and sunset; or
- Nighttime (or nocturnal) = occurring during the period after civil twilight ends in the evening and before civil twilight begins the next morning.

From the USNO website, we also obtained the precise times of sunrise/set and civil twilight begin/end calculated to the longitude and latitude coordinates of our camera trap location, and a meridian time of +7.24 h east of Greenwich Mean Time (GMT) (given our location is approximately 4°E of GMT +7 h; central meridian 105°E). Because our camera traps were originally set to CST (i.e., GMT +8 h; central meridian 120°E), we then corrected the times of independent events by subtracting 44 min from the image time stamps. Astronomical information regarding the moon was similarly gathered using the USNO data services.

## Results

Images of four vertebrate species were captured by our camera traps; they included those of *R. brelichi*, *M. thibetana*, the giant flying squirrel *Petaurista* sp., and the golden pheasant *Chrysolophus pictus*. From the images, we ascertained 24 independent events of our focal species that occurred on 21 days from April through October (except May) (Table 1). These events involved *R. brelichi* individuals of all age and sex classes, and they showed the monkeys active during different periods of the diel cycle. In particular, diurnal activity accounted for 18 of the 24 events (75 %), which were concentrated in early morning (i.e., from the beginning of civil twilight to ca. 30 min after sunrise) and late afternoon (i.e., from ca. 30 min before sunset to the end of civil twilight). The remaining six events (25 %) were considered nocturnal, and all but one (September 15, see below) appeared to be extensions of activity schedule beyond the periods of civil twilight. The monkeys’ activity events after sunset and before sunrise did not consistently correspond to the fraction of the moon illuminated. Moreover, based on the temporal distribution of events recorded, this or a nearby location was likely a nighttime sleeping site frequently used by *R. brelichi*.

**Table 1:** Date and time of independent events (*n* = 24) involving *R. brelichi* captured by two camera traps and the category of activity period as defined by the corresponding time of sunrise or sunset. All times are adjusted to reflect a meridian time base (E 108°45′, N 27°57′, +7.24 h east of GMT). See “Methods” for details

Date	Event time (h)	Activity period	Sunrise/set time^a^ (h)	Moon fraction^a^ (%)
04/22/11	1824–1833	Day → twilight	0528/1828	0, below horizon
04/23/11	0518–0519	Twilight	0527/1829	72
06/23/11	0527	Day	0504/1859	
07/21/11	1836–1839	Day	0516/1856	
07/24/11	1538	Day	0517/1854	
07/31/11	1839	Day	0521/1850	
08/01/11	0520–0541	Twilight → day	0522/1850	0, below horizon
08/25/11	1711–1728	Day	0534/1829	
08/28/11	1711–1752	Day	0536/1825	
08/29/11	0538–0550	Day	0536/1824	
09/15/11	0259–0300	Night	0544/1805	95
09/15/11	0605–0607	Day	0544/1805	
09/21/11	1829	Night	0547/1758	0, below horizon
09/22/11	0812–0854	Day	0547/1757	
09/27/11	0437	Night	0550/1751	0, below horizon
10/07/11	0746	Day	0555/1740	
10/07/11	1632–1715	Day	0555/1740	
10/08/11	0535–0536	Twilight	0555/1738	0, below horizon
10/10/11	0558	Day	0557/1736	
10/10/11	1701–1723	Day	0557/1736	
10/11/11	0529–0559	Night → twilight → day	0557/1735	0, below horizon
10/12/11	0541–0602	Twilight → day	0558/1734	100
10/13/11	1905	Night	0558/1733	98
10/14/11	1933	Night	0559/1732	95

^a^Data obtained from Astronomical Applications Department of the United States Naval Observatory (USNO). http://aa.usno.navy.mil. Moon fraction refers to the moon’s visible disk illuminated at the time the monkeys were active during twilight or at night

**Fig. 1:**
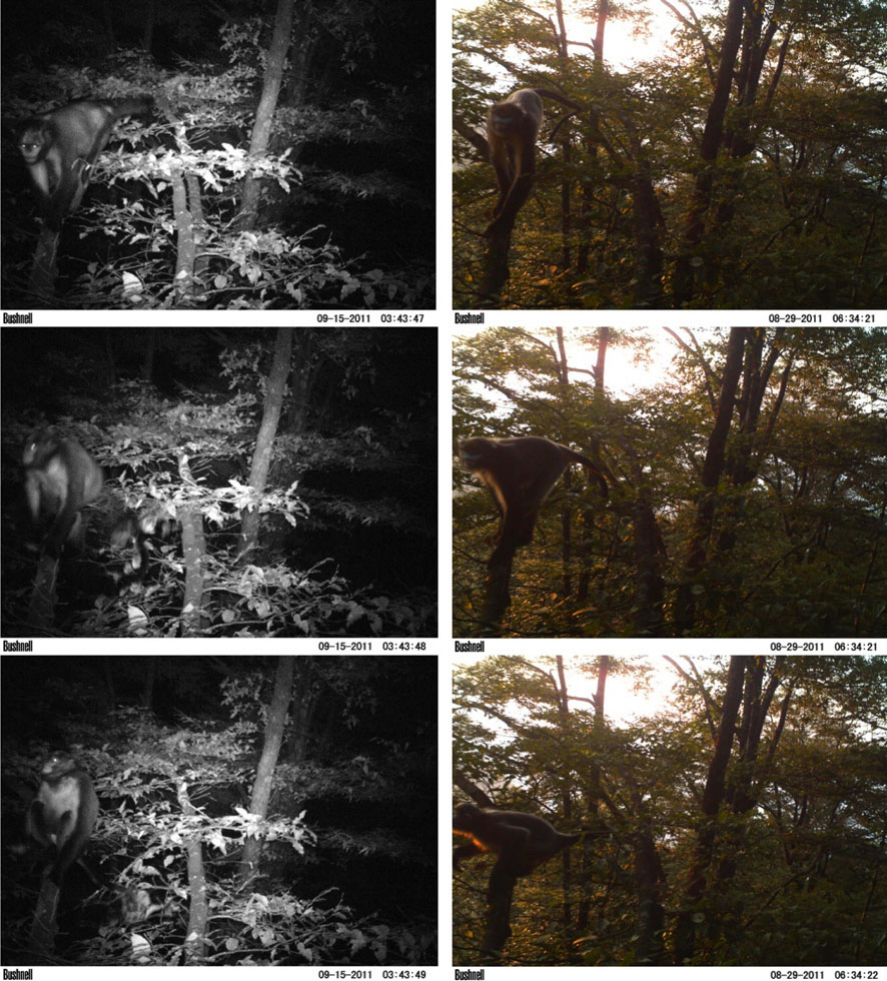
A comparison of nocturnal (*left column*) versus diurnal (*right column*) images captured by camera trap LT004 showing *R. brelichi* individuals moving through the beech forest (note: image time stamps reflect CST before correction; see “Methods”)

Of all the camera trap images, the most noteworthy and unexpected ones were captured on September 15 when *R. brelichi* individuals were observed to be active at 0259–0300 hours. Although only nine images were taken, they showed several monkeys, possibly members of a reproductive unit, moving at a steady pace along a fixed route in a manner similar to behaviors exhibited during daytime periods (Fig. 1).

## Discussion

The diel activity pattern of most living primates is characterized as either diurnal or nocturnal (Curtis and Rasmussen 2006). Since categorically diurnal and nocturnal primates differ significantly in their features of the visual system (Kay and Kirk 2000; Kirk and Kay 2004), this distinction has led to the assumption that primates are largely confined to a diel activity pattern due to constraints associated with a particular type of visual system. Among anthropoids, only members of the genus *Aotus* are known to be nocturnal (Wright 1989; Fernández-Duque 2003). Although nighttime activities of diurnal anthropoids have been alluded to in various accounts, in the absence of well-substantiated data, it is thought that these observations were anecdotal and diurnal primates normally remain inactive throughout the night (see review by Ankel-Simons and Rasmussen 2008). However, given that in nature primates experience varying intensity of illuminance throughout the 24-h day (Pariente 1980), a broad spectrum of visual capacity and behavioral flexibility would be expected and must not be overlooked (Ankel-Simons and Rasmussen 2008).

*R. brelichi*, according to conventional views, is believed to be exclusively diurnal. Overall our results concur with those obtained through direct observational studies that indicate *R. brelichi*, as other Chinese *Rhinopithecus* species, exhibits a predominantly diurnal activity pattern (e.g., Yang et al. 2002; Li 2009; Li et al. 2010; Xiang et al. 2010). However, our camera trap images provided irrefutable evidence that *R. brelichi* individuals are habitually active during twilight and night periods. We suspect that this extension of diurnal activity may reflect the species’ behavioral adaptations to living in a temperate environment where day length and food resources are highly variable across seasons. It has been shown, for example in baboons, that seasonal variation in daylight hours may pose an ecological constraint on the time available for a diurnal species to perform vital daily activities (Dunbar 1988; Hill et al. 2003). Thus, having an adjustable activity schedule may confer sizable advantages by allowing *R. brelichi* to increase foraging effort during favorable conditions, and thereby enhance their energy reserves as a buffer against adverse conditions later. We believe flexibility in their diel activity pattern, as well as diet (Niu et al. unpubl. data), may enable *R. brelichi* to survive the harsh winter conditions in Fanjingshan.

Furthermore, the ability of *R. brelichi* to extend activity into twilight and nighttime periods may be linked to other adaptive features of the monkey’s biology such as its visual system. It has been suggested that in mammals the visual system is shaped by the light regimes of the habitat in which each species lives (Veilleux and Lewis 2011). Thus, the visual system of *R. brelichi* may be optimized for functioning at low light (mesopic) levels as an adaptation to the forest of Fanjingshan where dense fog and low clouds are constant, resulting in a reduced light environment year-round. Indeed, there is much to be learned about the visual capabilities of *R. brelichi* and other *Rhinopithecus* species, and how their ecology has played a role in the evolution of their visual system or vice versa.

The observed diel activity pattern, derived from close to 300 days of 24-h continuous monitoring, was consistent throughout the study. As such, we believe this pattern may be the norm for *R. brelichi*. That *R. brelichi* (and possibly other temperate-dwelling *Rhinopithecus* species) exhibits a considerable amount of activity outside the typical diurnal observation schedule of researchers calls attention to methodological issues that arise from sampling bias. Granted that complete, full-day follows of free-ranging snub-nosed monkeys are labor intensive and difficult to achieve, we urge researchers to improve observational protocols and to reexamine the way in which we have characterized aspects of the monkeys’ behavior (e.g., activity pattern, time budget, diet, ranging, sleeping site use) that were predicated upon incomplete sampling days and/or unequal sampling effort.

To facilitate cross-site and/or cross-species comparisons, we recommend that researchers adopt a standard definition for day length to determine activity patterns and time budgets. Also researchers should convert all local times to a meridian-based time calculated using the latitude and longitude coordinates of their study site when evaluating activity patterns in relation to rise and set of the sun (as explained in our “Methods”). Particularly, adjustments of local times are paramount for studies conducted in mainland China, where only one official time zone (CST) is maintained even though the country occupies five time zones by breadth of longitude. Activity data collected based on CST without corrections, when related to the timing of astronomical events at the research location, can produce grossly inaccurate and noncomparable results. As an example, Xiang et al. (2010) reported absolute time budgets of *R. bieti* in Tibet based on calculations of day length that included periods of nautical twilight (i.e., the sun at 6–12° below the horizon; day length range 12–16 h). However, their observations of the monkeys were limited to “dawn to dusk” periods on days only with acceptable weather conditions (Xiang et al. 2010, p 653). Assuming their definition of “dawn to dusk” observation was from sunrise to sunset, their absolute time budgets for *R. bieti* would be artificially inflated by about 10 %, an amount equal to the two daily periods of nautical twilight during which no observations were made.

In short, using camera traps we discovered extended periods of diel activity of *R. brelichi* that were previously undetected through direct observation. We consider camera traps a valuable scientific tool for primate research, particularly for studies of diel activity patterns, because these devices offer 24-h continuous monitoring with minimal disturbance to the animals. With regular equipment maintenance and proper sampling protocols, camera traps can simultaneously record year-round data on the study species and other animals in the community. Although direct observation is essential in providing contextual information regarding the animal’s behavior, a remote camera-trapping approach certainly broadens not only our understanding of primate behavioral ecology, but also stimulates discussion about the need to further refine our observational study designs to reduce sampling bias.
